# Mars Colonization: Beyond Getting There

**DOI:** 10.1002/gch2.201800062

**Published:** 2018-10-25

**Authors:** Igor Levchenko, Shuyan Xu, Stéphane Mazouffre, Michael Keidar, Kateryna Bazaka

**Affiliations:** ^1^ Plasma Sources and Applications Centre/Space Propulsion Centre NIE Nanyang Technological University Singapore 637616 Singapore; ^2^ School of Chemistry Physics and Mechanical Engineering Queensland University of Technology Brisbane QLD 4000 Australia; ^3^ CNRS ICARE Electric Propulsion Team 1c Avenue de la Recherche Scientifique 45071 Orléans France; ^4^ Mechanical and Aerospace Engineering George Washington University Washington DC 20052 USA

**Keywords:** ethical considerations, legal considerations, mars colonization, space exploration

## Abstract

Colonization of Mars: As humans gradually overcome technological challenges of deep space missions, the possibility of exploration and colonization of extraterrestrial outposts is being seriously considered by space agencies and commercial entities alike. But should we do it just because we potentially can? Is such an undoubtedly risky adventure justified from the economic, legal, and ethical points of view? And even if it is, do we have a system of instruments necessary to effectively and fairly manage these aspects of colonization? In this essay, a rich diversity of current opinions on the pros and cons of Mars colonization voiced by space enthusiasts with backgrounds in space technology, economics, and materials science are examined.

## 1 Mars Colonization—Do We Need It?

Mars: Among other potential outposts, the Red Planet has always been shrouded by a veil of romanticism and mystery. Beyond an active target for space exploration, colonization of Mars has become a popular topic nowadays, fuelled by a potentially naive and somewhat questionable belief that this planet could at some point in time be terraformed to sustain human life.^1^ Indeed, the Moon, while very close, is small, barren and devoid of atmosphere. Life on the Moon base would not differ from that in the lifeless desert, with no hope of ever finding water. Other neighboring planets, such as hot Venus and gas giants Jupiter and Saturn, are no more suitable for human habitation.

**Figure 1:**
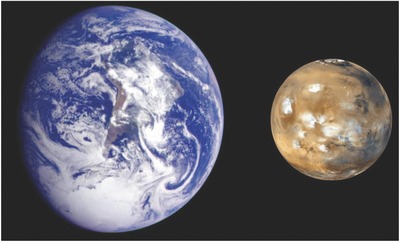
Composite image that shows the relative dimensions of Earth and Mars. The image of Earth was captured from the Galileo orbiter at about 6:10 a.m. Pacific Standard Time on December 11, 1990, when it was at a distance of ≈2.1 million kilometers away from Earth during the first of two Earth flybys on its journey to Jupiter. The image of Mars was captured by the Mars Global Surveyor in April of 1999. Image credit: NASA/Jet propulsion Lab.^5^

Mars, however, is a horse of a different color. With a mean radius of 0.53 of that of Earth,, i.e., a surface area nearly equal to the total area of dry land on our planet, and 0.38 of Earth's surface gravity (**Figure** **1**), Mars is thought to provide a potentially much more benevolent environment for the colonists from Earth compared to any other proximate planet. Moreover, promising results obtained by rovers and a low‐frequency radar installed on the Mars Express spacecraft have long sustained the belief that it might be possible to find undersurface and subglacial liquid water.^2^ Furthermore, similar to Earth, Mars is expected to have substantial mineral resource at and under its surface layer, with a recently confirmed evidence of metal ores and other vital mineral substances.^3^ Although no one has seriously demonstrated a practical means for the extraction and refining of these resources into useful products on Mars, a distant possibility of doing so is considered a principal point in favor of colonization. These features of the Red Planet have firmly cemented its status as an ultimate space colonization destination for near future,^4^ despite the obvious immediate challenges such as a dusty carbon dioxide‐rich atmosphere, the pressure of which is reaching only 0.09 atm.

Intense efforts by the world's space agencies and more recently, private enterprises have brought us ever closer to having broad technical capabilities to transport a small number of colonizers and equipment to Mars. These capabilities have been discussed in detail in several comprehensive review and opinion articles that describe various opportunities and challenges facing the Mars settlement program.^6^ Proponents of Mars colonization consider present space technology as nearing the stage when it will be able to provide the necessary level of reliability and efficiency required for the one way journey from Earth to Mars. Indeed, a recent example of successful firing of thrusters on Voyager 1 after 37 years of space operation^7^ attests to our ability to overcome such significant challenges of spacecraft development^8^, ^9^ as longevity, reliability, and operational readiness decades after launching. Ongoing advances in nanotechnology and materials engineering enhanced reliability and expanded functionality of contemporary electronics and robotics while reducing device mass, volume, and power consumption.^10^ The affordability of small space assets has enabled greater exploration of space, allowing space agencies, universities, and commercial players to collect vital information about extraterrestrial environments in which space assets and living subject will be required to operate, guiding and informing the development of colonization programs.^11^

Is it time to go extra‐terrestrial? Mars One program has beenoperating since 2012 and, considering the present level of financial and public support, it is very likely to continue.^12^ Falcon Heavy, presently the world's most powerful rocket capable of delivering about 17 tons to Mars surface, was successfully launched on 6 February 2018, demonstrating its capacity to deliver payloads within the framework of Mars One program.^13^ In parallel, efforts are made to develop plausible geodynamic scenarios and define relevant parameters,^14^ including ambitious ideas of future Mars terraforming.^15^ Materials suited for Mars‐oriented applications and operation environments are also under active development.^16^ Technical aspects of these projects are described in numerous roadmaps and system architecture description documents.^17^ To some, these developments provide confidence that it will indeed be possible to begin colonization of Mars within our lifetime, at least from a technological point of view. And there is certainly no lack of volunteers keen to take on the challenge of a 7 month long one‐way journey to the Red Planet. Indeed, since Mars One's call, thousands have applied and about 100 have been preselected as potential candidates to make up the first crew of four astronauts to be sent to Mars in 2031.^18^

**Figure 2:**
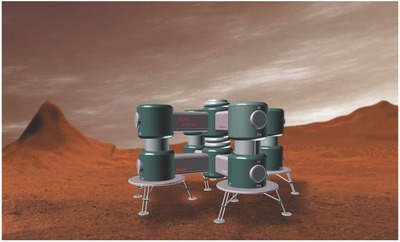
Modular Martian settlement (artistic representation). Several alternative modular concepts have been proposed, including one by Mars One.^11^

Upon reaching the surface, the astronauts will be expected to establish a permanent settlement on Mars, collecting vital data and conducting experiments, with the clear expectation never to return to Earth again (**Figure** **2**).^19^

Settlement of Mars—is it a dream or a necessity? From scientific publications to public forms, there is certainly little consensus on whether colonization of Mars is necessary or even possible, with a rich diversity of opinions that range from categorical It is a necessity!^20^ to equally categorical Should Humans Colonize Other Planets? No.^21^ A strong proponent of the idea, Orwig puts forward five reasons for Mars colonization, implicitly stating that establishing a permanent colony of humans on Mars is no longer an option but a real necessity.^20^

Specifically, these arguments are:

1. Survival of humans as a species;
2. Exploring the potential of life on Mars to sustain humans;
3. Using space technology to positively contribute to our quality of life, from health to minimizing and reversing negative aspects of anthropogenic activity of humans on Earth;
4. Developing as a species;
5. Gaining political and economic leadership.

The first argument captures the essence of what most space colonization proponents feel—our ever growing environmental footprint threatens the survival of human race on Earth. Indeed, a large body of evidence points to human activity as the main cause of extinction of many species, with shrinking biodiversity and depleting resources threatening the very survival of humans on this planet. Colonization of other planets could potentially increase the probability of our survival.

*While being at the core of such ambitious projects as Mars One, a self‐sustained colony of any size on Mars is hardly feasible in the foreseeable future. Indeed, sustaining even a small number of colonists would require a continuous supply of food, oxygen, water and basic materials. At this stage, it is not clear whether it would be possible to establish a system that would generate these resources locally, or whether it would at least in part rely on the delivery of these resources (or essential components necessary for their local production) from Earth. Beyond the supply of these very basic resources, it would be quite challenging if not impossible for the colonists to independently produce hi‐tech but vitally important assets such as medicines, electronics and robotics systems, or advanced materials that provide us with a decent quality of life. In this case, would their existence become little more than the jogtrot of life, as compared with the standards expected at the Earth?* 22

This brings us to the second argument—in order to deliver any positive change to the quality of life of humans on Earth, the question of Mars colonization should not only be about survival but also about development if it is to present a viable alternative to our current existence. Such development is inherently linked to the availability of local resources required to sustain life, which is in turn reliant on the availability of instrumentation and equipment necessary for their discovery, extraction and refining. There is little doubt that in early stages of Mars colonization, the greatest fraction of the payload delivered to Mars will be dedicated to equipment needed to provide critical infrastructure and sustain the most fundamental needs of the colony, and not scientific instruments for greater Mars exploration. However, it should be noted that with recent advancements in miniaturized, energy‐efficient electronic and robotics devices, it may in principle be possible to deliver a highly functional yet compact automated laboratory to Mars. A recent breakthrough discovery of (possible) ancient “building blocks of life” made by Curiosity rover greatly supports this notion.^23^, ^24^ Where Curiosity accommodates only 6.8 kg of scientific instruments, the scientific capabilities of a high‐tech laboratory delivered by one of Mars One landing units solely dedicated to such a mission (i.e., not carrying humans and related resources) could be quite considerable.

The third argument relates to technological advances related to space exploration, specifically how technologies that we may develop in our effort to colonize Mars may find their way into our daily life and deliver unintended benefits. As an example, Orwig points to the image analysis algorithm originally developed for extracting information from blurry images received from Hubble Space Telescope. After the technology was shared with a medical practitioner and as a result applied to medical images, such as X‐ray images, it enabled more accurate visualization of breast tissues affected by cancer, and subsequently led to the development of a minimally invasive stereotactic large‐core needle biopsy.^25^ In a separate study, the sequencing and analysis methods developed by NASA to detect and characterize bacterial species on spacecraft to effectively prevent contamination of other worlds with Earth's biota was used to study the link between microorganisms in breast ductal fluid and breast cancer.^26^

Finally, the fourth and fifth arguments refer to Mars colonization as an opportunity for humans to grow as a civilization, actively changing the way in which we interact with and exploit our environment. Indeed, in this aspect we can (following Pyne) consider Mars colonization as a kind of cultural invention.^27^ Looking back to the Age of Exploration, could the exploration of near‐Earth space together with the Mars and Moon colonization be judged as unavoidable and intuitive continuation of processes started at the dawn of human civilization? Some would argue so, as Shiga points out: “All of the space shuttles – and the ill‐fated Mars rover, Beagle – were named after famous sea vessels.”^28^ To many, such a deep attachment to rich history of nautical exploration certainly confirms this hypothesis.

At this point, it is not entirely clear what opportunities and challenges living on Mars will present, and how we as a species would respond to these, but there are certainly calls to embrace innovation and sustainability as the only means to ensure the quality of life for generations to come. Yet, who will oversee and enforce these ideals? Indeed, at its early stages of settlement, the small colony is likely to be composed of altruistic, selfless, technologically savvy individuals who may thrive in an equitable and libertarian society and may be prepared to sacrifice individual desires and benefits for the greater good of the group. However, it is far less likely that such a system can be sustained once the population of colonists grows to thousands and millions and becomes more diverse. Inevitably, a socioeconomic and political order will emerge, and it is likely to be different from the initial system. Would it be possible not to repeat mistakes that we have made when colonizing continents here on Earth?

As we race toward realizing technical aspects of Mars colonization, these and other questions certainly warrant further investigation and discussion. Should we spend a tremendous amount of intellectual, financial and material resources on a distant dream over addressing immediate and highly pressing problems that threaten our very existence on Earth? And is having technological capacity to get there a good enough reason for colonization? In the remainder of this Essay, we will briefly introduce a number of opinions on these issues from stakeholders and space science enthusiasts with diverse backgrounds.

## 2 Legal Considerations

Right now, the Outer Space Treaty^29^ is the main document that governs international cooperation and intercommunication around space and other celestial bodies. While the Outer Space Treaty does not prohibit colonization of Mars, building a permanent colony on the surface of Mars will certainly call for the development of a new system of laws and regulations, which potential colonists would be required to abide by, and which would take precedence over any laws and regulations governing their country of origin. As already mentioned earlier, this may be possible for a small group of like‐minded individuals with common values. Yet, as the colony grows and becomes more diverse with respect to customs, beliefs, traditions and ways of thinking, this may become increasingly challenging. Will it be easy for all interested parties to outline and accept such “Mars constitution”? The success of this endeavor is at the very least questionable, since the major space‐faring nations could not even sign off on The Moon Treaty.^30^, ^31^ Now, we see efforts by the United Nations to initiate the coordination of space‐related activities,^32^ along with active public debates on this problem.^33^, ^34^ Below we outline some specific legal considerations raised in the recent publications on the topic.

### 2.1 Do Earth Laws Apply To Mars Colonists?

A set of fundamental questions regarding governance on Mars was formulated by a known proponent of Mars colonization, professor of space law Dunk and discussed by Fecht in her paper *Do Earth laws apply to mars colonists*?^35^, ^36^ Since the demise of Soviet Union, the funding for many national space programs, such as NASA, has not experienced a significant increase, thus keeping the available financial and human resources at a relatively stable level.^37^ This provided private companies, such as those led by Musk, an opportunity to emerge and eventually become critical players in space exploration and colonization. Signed in 1967 when space exploration was dominated by nations and not private companies, the current Treaty does not preclude the latter from travelling to Mars, as pointed out by Dunk.^35^, ^38^ According to his interpretation, private companies can deliver payloads to the surface of the Red Planet and settle on it permanently. We should mention here that the Outer Space Treaty has an international character and does not list specific regulations. However, it does prohibit potential settlers from launching weapons of mass destruction and defining land ownership. These laws are modeled on those on Earth, where deployment of any rocket into space requires multiple levels of authorization at the government and international levels, with the specifics defined by the nature of proposed activities in space. For instance, the launch and operation of a telecom satellite requires approval by the Federal Communications Commission.^39^ As global activities in space increase and the number of private enterprises engaged in space exploration grows rapidly, we should expect significant changes in the active regulatory environment in the near future.

While Mars One project has an essentially international character, it still may be bound by the US laws depending on the level of participation of American companies in the project. Mars One is known to rely on third‐party vendors for heavy rocket platforms, with the SpaceX Falcon Heavy, and possibly SLS^40^ and BFR^41^ being the only realistic options in the near future. Regardless of the country from which it is launched, the rocket produced by an American company will be regarded as an American ship, and, following a very similar approach that governs the behavior of sea‐fairing ships, the space ship would have to abide by the laws of the US legal system. In yet another analogy to the maritime system, the surface of Mars would not belong to any particular country or entity, just as international waters do not belong to any nation. Indeed, even upon reaching the surface of Mars and disembarking the ship, the colonists would be expected to follow the rules of the country that has jurisdiction over their ship. Furthermore, any permanent outpost would be expected to develop an independent governing system, yet the nature of this system is debatable.^35^

Recent important efforts to develop an updated legislative system, such as U.S. Commercial Space Launch Competitiveness Act^42^ and Act of 20 July 2017 on the exploration and use of space resources^43^ aim to go beyond the Outer Space Treaty. These two sets of laws postulate that space resources can indeed be used and exploited by private companies and investors.

On one hand, the early system may capture and be driven by the altruistic nature of early settlers. At the same time, those first settlers will also be subject to a harsh environment, very limited resources and extreme social isolation and uncertainty, potentially necessitating a system that is more hierarchical and rigid. As the colony grows, an increasingly complex legal system may emerge on the back of multifaceted socioeconomic processes, yet it is still likely to be affected by scarcity of resources and a psychologically challenging living environment. As such, it would be necessary to create an authority that would enforce these laws, ensure their effectiveness, and manage those situations where these laws are challenged. Indeed, the latter is inevitable, both because the laws must evolve to adequately reflect a dynamic socioeconomic and technological environment, as well as for the reasons of human nature, where one has a propensity to take advantage of others.^44^ With these factors considered, it is difficult to imagine that modern legal systems we currently have on Earth would be appropriate to govern the life on Mars.

### 2.2 Sovereignty

The question of sovereignty of permanent colonies on the surface of Mars and, possibly, in the Martian orbit is one that at present is not well articulated or defined in the current version of the Outer Space Treaty. At present, it is not possible for a nation or an entity to lay claim of sovereignty over a celestial body or any artificial habitable human outpost, such as a space station. However, it is not clear whether this principle can be upheld as we move into advanced stages of peaceful space colonization, such as that of Mars. Multiple models have been proposed. For instance, Bruhns and Haqq‐Misra suggest a so‐called “pragmatic approach to sovereignty on Mars”, where they explore the benefits of adopting a policy that balances “bounded first possession” against mandatory planetary parks. The former would allow nations to hold legal jurisdiction and exclusive rights to economic benefits derived from a parcel of land, whereas the latter would enable protection of areas of natural, ecological, scientific or cultural significance for the benefit of global community. The proponents of this approach assume that the private property rights‐based economy is the best option for the development of Mars society, and it may indeed be so for the advanced stage of Mars colonization. The relationships between such colonies would be managed diplomatically in accordance with international treaties, and if necessary, the resolution of conflicts may be administered by a formal commission, agency or tribunal with representatives from Mars colonies. Indeed, Bruhns and Haqq‐Misra suggest establishing a Mars Secretariat, the role of which would be to formally enable and facilitate diplomatic communication between interested parties. Broadly, this approach reflects the general principles of the Outer Space Treaty, while providing a more practical model for the management of resources and economic benefits that can be derived from Martian colonies by introducing changes to the non‐appropriation and province of mankind principles.^45^ Clarification of the rules that govern the derivation and use of Martian resources by nations and private entities is essential to avoid conflict between future colonies at the stage when resource extraction and exchange would become possible.

### 2.3 Human Rights

It remains a subject of debate to which extent human rights can be ensured when one considers establishing a permanent colony on Mars. Indeed, there is little doubt that the journey first colonists undertake would be a “one‐way” endeavor. That is, they will have no physical means of ever returning to Earth. The romanticism of being the first to plant a step on the surface of Mars and the overall sense of this effort as being a giant leap for humanity has led to many expressing their strong interest in taking part in the project. At present, these enthusiasts are prepared to sign over their most basic rights of free choice of residence, profession, right to adequate medical treatment and many others for this opportunity. But do we have a legal and in fact a moral right to knowingly subject others to such a life, even with their consent? Below are examples of three different considerations that could play a significant role in such a discussion.

In the first scenario, let us consider a physical illness or mental breakdown that would lead to the volunteer requesting to withdraw their consent to be part of this journey. Would the organizers have a legal right to enforce the original agreement when the participant invokes their human rights and requests their return to Earth through a legal mechanism? Indeed, let us imagine an Earth‐based experiment where a person is subjected to the life‐term isolation in a relatively good, yet significantly restricted environment, e.g., an Antarctic base. The volunteers would document their consent to spend the rest of their lives under the experimental conditions, however at some stage would change their mind and withdraw their consent, requesting that they are removed from the experiment. Would the legal system and public opinion support the company in their choice of forcefully retaining the volunteer under experimental conditions in accordance with their original properly documented consent agreement? It is difficult to imagine that they would, as this would violate the basic human rights of the individual. If so, who will be financially responsible for retrieving these volunteers and returning them to Earth? This situation merits careful legal consideration prior to such a flight.

Let us consider the second scenario where the volunteer legally challenges the agreement on the basis of failure of the entity to comply with promises and conditions of the original agreement. It is hardly difficult to imagine that the reality and specific conditions of life on Mars will be different from even our best estimates and expectations. If these differences are quite substantial, mission participants may give legal grounds for a complaint. Considering that the first wave of colonizers may remain formally under jurisdiction of their country of origin, they would likely retain the full rights to call on their respective legal system and body of authority to protect their interests. Not only can it develop into a complicated legal case for which no precedent exists, it may potentially force the entity in question to take certain measures and as a result jeopardize the success of the mission or program. It is therefore likely that a range of legal and financial obligations will be placed on travel organizers to deal with such complaints. While it may be impossible to retrieve and return colonists to Earth during early stages of colonization, technological advances may eventually make such missions technically possible but prohibitively expensive endeavors. In the worst case scenario, a court's order may be issued, with the enforcement machinery ordering the organizers to take actions on starting the “return project.”

The third scenario that we are going to consider relates to the rights of children born on Mars. Reproductive rights are at the core of many legal systems, and as such would apply to colonists that settle on Mars. These include the right to decide on the number and spacing of offspring, and the right to attain an appropriate level of sexual and reproductive healthcare. Thus, one would expect children to be born on Mars. In fact, some argue that these children would be critical for the long‐term success of the colony as they should be better suited, both physically and psychologically, to the unique living conditions of the Red Planet. They would also be the driving force for the growth and development of the colony, as one could hardly expect all of its inhabitants to be shipped from Earth.

Again, drawing parallels to current legislation on Earth, children born to parents of particular nation would likely inherit the citizenship of their parents, able to exercise the rights of that particular legal system. This in itself may represent a challenge, since given a very small size of the colony, parents may belong to different systems, each having its own idea of how rights of children should be protected. Even within a single system, it is rather challenging to envisage what instruments and mechanisms will be put in place to protect the rights of children on Mars. Similarly, what authority would manage the relationships between children and their parents, or between parents in the case of their separation and divorce? Furthermore, community and family support are critical for families during the time of hardship or conflict, and children on Mars would most certainly lack this safety net.

However, before we even consider potential threats to children's health and wellbeing, at which point would standards of living on Mars reach a minimum acceptable level of health and safety for the reproduction to become ethical? Furthermore, even if we have sufficient technical capability to maintain a decent quality of health and safety of Mars, we would certainly not be able to provide the same degree of choice, e.g., in terms of education or profession, to these children as those available to children on Earth. What legal rights would these children have to request their relocation to Earth? Indeed, are we prepared to rationalize the life of isolation and restriction these children would have to endure—the life they have never consented to. Could—or should—they be considered by the relevant authorities as kids that are retained under what most describe as rather harsh or even inhumane living conditions? Article 6 of the Convention on the Rights of the Child states that “*Governments should ensure that children survive and develop healthily*”; article 24 states: “*Children have the right to good quality health care – the best health care possible*.”; and Article 27 requests an adequate standard of living.^46^

Apart from these legal considerations, ethical considerations related to the reproduction on Mars may be a significant issue, with some opinions presented in the following section.

We should also mention that these considerations are not exclusive to Mars. For instance, any woman of childbearing age is required to undergo mandatory pregnancy testing before she is allowed to take part in missions that involve extreme conditions, such as an expedition to Antarctica under the U.S. Antarctic Program.^47^ And this is considering that it is possible and comparatively easy for the woman to be retrieved from the expedition in the case of medical emergency. In fact, the very nature of such expeditions is temporary, and all members are expected to return home within a relatively short period of time. This is in stark contrast to expeditions to Mars, where participants are expected to be responsible for their own healthcare and wellbeing and have to exclusively rely on their own human and technological capacity permanently.

Further, as the colonist population grows, it is likely that homicides, robberies, and other criminal actions will occur. These events would necessitate some form of criminal justice and punitive system to be established on Mars at the further stages of colonization to prosecute and deliver punitive measures to offenders. Yet, with every pair of hands and skill set being critical for the success of the colony, to which extent would conventional corrective actions be feasible within the unique environment of a space colony? Therefore, the question remains: which laws would apply?

### 2.4 Abortion

The issues around abortion are closely related to those of human rights, yet often are considered separately due to their intimate relationship to cultural and religious beliefs of different groups of people. Presently, in many nations abortion is viewed as a right of women and a matter of private choice, whereas in others it is legally considered a crime. Considering that a colony on Mars may comprise representatives from different cultural and religious belief systems, it may be difficult to design a policy that would be acceptable to all. Nevertheless, some expect the abortion policy of a Martian colony to be more liberal compared to that on Earth, particularly when it comes to choice based on medical grounds. Indeed, pregnancy termination may be required in instances where pregnancy endangers the life and health of the woman. Similarly, it is difficult to imagine that harsh Martian conditions would be suited for children with severe debilitating medical conditions simply due to the complete lack of infrastructure to afford them a decent quality of life. Caring for such a child would also be quite consuming in terms of time, human and physical resources, potentially redirecting these resources from activities critical to colony survival and development. Beyond these considerations, it is not clear what other medical and biological challenges of reproduction and living on Mars would inform the abortion policy.^48^ It is likely that it would emerge and evolve in parallel with our understanding of what life on Mars would entail.

## 3 Ethical Considerations

Ethical considerations and issues around Mars colonization can be intuitively separated into two significantly different groups of questions, namely:

• Ethical considerations with respect to humans, both colonists and people of Earth, and
• Ethical considerations towards Mars itself, including possible extra‐terrestrial life.

Both are important, and below we will outline some opinions, sometimes controversial, around the ethics related to Mars colonization.

### 3.1 General

Decades of intense efforts by thousands of people and billions of dollars in funding would likely culminate in sending a small group of four to five individuals on a one‐way trip to Mars. The success of the mission would depend on how well these individuals can work together to handle an environment that is extreme both physically and psychologically. It is therefore likely that the greater good of the group and thus the success of the mission would supersede that of individuals, a pattern of behavior that is not typical of people in their natural habitat due to the differences in judgment of values. For this reason, a framework of decisions that benefit the group over an individual is likely to be defined, with considerations over such personal matters as termination of defective fetuses, euthanasia of individuals suffering from incurable debilitating conditions, and the act of sacrifice of individual life for the sake of the colony.^48^ There are evident similarities with sacrifices made by individuals during exploration endeavors during the Age of Discovery on Earth.^49^ Yet, these historic experiences also tell us that it is virtually impossible to foresee and control the behavior of individuals and groups when subjected to extreme survival situations. From this perspective, it is difficult to say what control if any flight organizations would have over the life of the colony.

NASA Human Research Program aims to study the risks associated with space flight over extended periods of time. Isolation and closed environment are some of the known factors to cause psychiatric distress.^50^ These medical conditions can be as damaging to the overall health of the space traveller and success of the mission as effects of space radiation, bone and muscle loss, and treatment of sustained injuries. Studies involving individuals and groups subjected to isolation have shown that social isolation stimulated brain activity toward short‐term self‐preservation, characterized by enhanced implicit vigilance for social threats even in the absence of thereof. Isolation also promoted more abrasive and defensive behavior in individuals, which may negatively affect the social dynamics of a small crew, even to the extent of mission sabotage. These issues, both psychological and physiological, are difficult if not impossible to address, and are independent of cultural, religious or educational background. Knowing the significant risks that cannot be mitigated, how can we make this venture ethical? Of course, all participants will be made fully aware of all known risks associated with the mission, and asked for their consent. However, does informed consent immediately make it ethical? Before we can answer this question, a wide discussion involving stakeholders and general public is certainly necessary to draw a line of what sacrifices are we prepared to take to make space travel and colonization a reality, and whether the benefits of spacefaring truly outweigh all the costs and risks of such adventures.^51^

### 3.2 Human Reproduction—Ethical Considerations

Biological and social challenges of human reproduction at a permanent Mars base are one more serious consideration that could potentially undermine the success of extra‐terrestrial colonization.^48^ Studies of human population dynamics on Earth suggest that the success of settlements on Mars would be inherently linked to the ability of early settlers to produce a certain number of viable offspring as these would be critical for the survival and growth of the colonies as self‐sustained entities. Resettlement of individuals from Earth should provide the foundations for a colony, yet overtime should become only a secondary source of residents. According to Impey, a population of at least 5000 is required to ensure long‐term survival of an extra‐terrestrial colony.^52^ It is difficult to estimate the physical and financial resources that would be required to realize a colony of such a size on Mars, and without a doubt would take a number of decades from the first successful mission. Indeed, the SpaceX Interplanetary Transport System is expected to carry only a small number of passengers, with a real possibility that not all of these individuals would be able to survive the 7–9 month‐long journey and the initial period of settlement and adaptation on Mars. This is not to say that such large‐scale transportation missions are not being seriously considered, and overtime it is expected that these missions would become more affordable and safer.

It is also difficult to predict the number of individuals that would be prepared to travel to and live on Mars. Indeed, on Earth, migration is an ancient phenomenon, yet it often carries significant negative impacts on health and mental well‐being of both the migrants and the local population.^53^ This is often due to a number of factors, such as being not fully prepared to commit and adjust to the new environment, differences in cultural, social and legal norms, and others. Differences in the physical environment may also negatively affect the physical health and wellbeing of newcomers. From this perspective, individuals that are born and brought up within the colony may be better suited to physical and psychological conditions of Mars, and as such may be better prepared to embrace life as part of a colony.

However, realizing sustainable human reproduction on Mars may not be without its challenges. For one, the number of available individuals would be small, affecting genetic diversity and increasing the likelihood of recessive genetic disorders. It will therefore be essential to enforce genetic, epigenetic and phenotypic screening of potential parents prior to conception, and then monitor the health and development of the fetus across all stages of the pregnancy to anticipate and minimize the risks of offspring being born with debilitating conditions. In addition to a legislative framework surrounding termination of fetuses that are unlikely to result in a birth of a healthy child,^48^ the same body of arguments may be applied to define which members of the colony should be encouraged or actively discouraged from having offspring.

Another consideration is the potential threat to the entire colony that may arise as a result of reproduction. Indeed, the success of the mission during the journey and within the early stages of the settlement is inherently linked to efficient utilization of human and physical resources. Bearing a child would divert some of these critical (and very limited) resources from the needs of the crew and activities associated with the survival of the crew during the flight and on the surface. Clearly, this warrants further investigation to have a better understanding of all the challenges and opportunities presented by pregnancy and child bearing on health and wellbeing of the crew during early space missions.^54^, ^55^

Finally, the general question of the growth of population in Mars colonies could be an issue. Indeed, will “native” Mars colonists accept newcomers, especially if living conditions are hard? After which period of time and at what stage of the colony development could they claim the land, or Mars in its entirety, as their property? In short, at which point in time would they come to consider themselves as the real Martians?

### 3.3 Social Isolation and no Privacy—Rolled Into One

Considering the aforementioned moral and ethical challenges that would need to be reconciled before we venture to Mars, it is evident that the definition of value of human life, choice, and privacy may take quite a different meaning on Mars to that on Earth. From this, one can conclude that the moral and ethical belief system of Martian society would be different to that of their Earthly counterparts, yet these individuals will still be subject to laws of the nation of their citizenship, at least at early stages of colonization.^48^ Furthermore, the role of these early settlers is to explore their environment and its effects on human body and social structure. It is likely that these individuals will be subject to ongoing monitoring and surveillance, which can have serious detrimental effects on their mental and physical health. These can exacerbate mental health consequences of physical confinement and social isolation, causing excessive suspiciousness, abrasiveness, stress, depression, and fatigue.^52^

In his “Those sent to live and die on the red planet face untold risk of mental illness,” Chambers explores a scenario of what might happen when the psychological pressure of isolation and a complete lack of privacy tip the colonists over the edge of mental breakdown, prompting them to temporarily or even permanently sever these surveillance channels.^56^ There is little published research on the extent of extreme psychological burden Mars colonists would be subjected to as part of, e.g., Mars One mission. Yet understanding these would be necessary to inform the selection of prospective participants. For example, resilience, adaptability, curiosity, creativity, and ability to place trust in others were listed as key traits for applicants to Mars One program, yet it is not clear how these will be measured and evaluated, and which traits will be deemed as not appropriate for the mission. Furthermore, it is not evident whether these traits are considered critical for minimizing the likelihood of one developing a mental illness because of prolonged social isolation or whether they are predictors of better emotional stability. Regardless of their attitude, there is little doubt that some of the selected individuals will develop mental illness, since even the most experienced members of space crew develop symptoms of anxiety, depression and apathy after extended period of time in space. This is despite decades of training, and a clear understanding that they will return to Earth upon completion of the mission.

**Figure 3:**
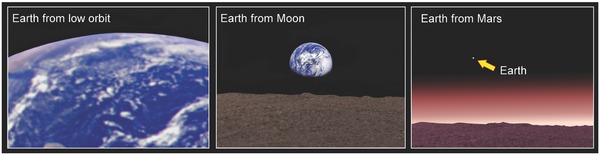
Social isolation on Mars would be a great source of stress to the colonists. While Earth is in close proximity to the International Space Station (ISS), it becomes a remote planet when seen from the surface of the Moon and is desperately lost in space when observed from the surface of Mars. Earth photos credit: NASA/Jet propulsion Lab.

According to an expert in psychology of space exploration and a Principal Investigator on several NASA‐funded and ESA‐sponsored international psychological research projects Kanas, upon departing Earth on their one way journey to Mars, the crew are likely to experience extreme homesickness, boredom, and loneliness (**Figure** **3**), which can lead to anything from dysphoria to psychosis and suicidal thinking. Upon reaching the surface of Mars, the colonists will swap their small spacecraft for an equally restricted base environment (≈50 m^2^ per person) in which they would spend the vast majority of their time.^57^ This is because Martian atmosphere is unbreathable for a human, with ≈96% CO_2_ and <≈1% of O_2_, as opposed to <≈1% CO_2_ and 21% of O_2_ on Earth. The surface temperature on the Red Planet averages −55 °C (218 K), reaching a peak of ≈20 °C at the equator, and a low of ≈−153 °C at the poles. There is evidence that the enjoyment of natural outdoor environment and diverse sensory experiences reduces stress and improves mental health.^58^

*“Worse still, imagine a mission that has no Third Quarter. Or no quarters at all! Step forward Mars One. During such a mission, our contestants will be without any of the psychological buffers that every crew has had since Gagarin. No real time interaction with family. No instant access to mission control. No option of returning home”*—writes Erik Seedhouse.^59^

### 3.4 Advocacy for Mars—Is It Ethical at All to Colonize It?

One of the strongest arguments in favor of Mars colonization is the survival of humankind in the case of a global event that would significantly compromise or even destroy modern civilization, e.g., a global catastrophe that would make Earth no longer habitable for our species. Having a distant outpost on Mars would allow us to escape the consequences of such an event, and persist as a species. Yet our history tells us that colonists, no matter how responsible, would inevitable affect the environment they colonize. Although our chances of discovering intelligent life in space are quite low,^60^ there remains a possibility of discovery of abiogenesis on Mars. Such a discovery would have tremendous scientific and philosophical significance, providing a second, potentially novel example of biochemistry and evolutionary history, and providing evidence for the phenomenon of life being spread across the universe. And most importantly, as an astrobiologist McKay points out, this will be an ultimate proof that extra‐terrestrial life in higher forms is possible.^61^

However, what if the native life, no matter how primitive, is incompatible with out notion of what Mars should become in order to accommodate human life. While the environment of Mars is certainly harsh, it may still support extremophiles. Indeed, on Earth there are a number of examples of microorganisms that can withstand extreme temperatures, e.g., *Pyrococcus furiosus* and *Pyrolobus fumarii*, pH, e.g., *Natronobacterium* and *Clostridium paradoxum*, pressures, e.g., *Pyrococcus* sp., and radiation conditions, e.g., *Thermococcus gammatolerans*. If native life is discovered, should it be preserved and protected? Would it even be possible to discover and recognize these most probably microscopic organisms before changing their environment? Currently, to reduce the possibility of contaminating other worlds with microorganisms from Earth, efforts are made to ensure that both the robotic and human exploration of extra‐terrestrial environments is biologically reversible. It should therefore be possible to reverse any possible contamination of Mars if signs of abiogenesis are detected.

However, should we in fact protect this life? On Earth, microbial decontamination is widespread and in fact critical to food safety, healthcare, and in many instances our survival. At which point our own need for survival would give us permission to threaten theirs?^62^ If life on Mars is discovered, it may be possible to consider other celestial bodies, e.g., the moons or sufficiently large asteroids, yet at present point in time, Mars appears to be the humanity's best option.^63^

Even in the absence of native life forms, there is an obligation for the colonists to attempt to preserve where possible the unspoiled alien environment, to ensure our sustained survival on the Red Planet. Yet, it is unclear how these ideas of preservation of native environment would balance those of terraforming of Mars through global engineering to make its surface and climate hospitable to humans. If attainable, the latter would make colonization of Mars safer and more sustainable.^64^ Clearly, it would not be possible to transport all the raw materials required for sustained growth and operation of a colony from Earth. Thus, these would have to be extracted from Martian environment, inevitably changing it.

*“Do we deserve to become multi‐planetary? Let us become productive participants in the glorious dance of life. If we can dream of the insurmountable task of becoming multi‐planetary, then surely we can fathom expending the energy, resources and willpower that come with making mindful purchase and waste decisions. If we can succeed in preserving our current planet and its ecosystems, we save human consciousness and the integrity of our values. As Elon Musk describes his desire to keep the “light of consciousness” alive, I press that we also ensure it's brightly illuminated and worthy of traversing this magnificent universe,”* writes Shivika Sinha.^65^

Apart from moral aspects surrounding the protection of possible life on Mars, there are potential legal issues directly related to preservation of Martian environment. Indeed the Outer Space Treaty does not directly prohibit colonization of Mars, but it explicitly states that *“States Parties to the Treaty… pursue studies of outer space, including the Moon and other celestial bodies, and conduct exploration of them so as to avoid their harmful contamination and also adverse changes in the environment of the Earth resulting from the introduction of extra‐terrestrial matter and, where necessary, shall adopt appropriate measures for this purpose”* (Outer Space Treaty, Article IX^29^). Yet, one can hardly imagine Mars colonization to proceed without any significant effect on the planet, let alone Mars terraforming, a process that assumes a significant and irreversible transformation of the environment. In this context, the Outer Space Treaty prescribes international consultations to take place before proceeding with such a project. Yet, what would be considered a harmful effect? It is definitely a gray area with considerable room for interpretation. Moreover, The Committee on Space Research (COSPAR) has also issued the Planetary Protection Policy, designed to regulate biological and other types of contaminations of celestial bodies stemming from human space exploration efforts.^66^

## 4 Consideration of Resources

Finally, let us consider the financial and resource aspects of Mars colonization projects and Mars exploration in general. Could it be a lucrative venture, or will Martian colony become a “groundnut scheme” of our generation?

In recent years, the idea of sustainable space economy where nations and private enterprises may derive financial benefits from extraction and utilization of extra‐terrestrial material and energy resources has gained notable attention. The proposed activities range from mining asteroids and the Moon to space tourism and development of large‐scale on‐orbit platforms that could offer a range of technical capabilities. Development of scientific research stations on the surface of large asteroids, the Moon and Mars are also considered.^67^

These are very ambitious yet tremendously costly projects that are highly risky from an investment point of view. What is the current financing model for Mars One project? The realization of Mars One mission to bring humans to Mars is managed by the not‐for‐profit Mars One Foundation, which relies on established aerospace suppliers to develop and assemble its aerospace hardware systems. At present, the cost of delivering a crew of four colonists to the surface of Mars is estimated at about US$ 12 billion with the cumulative cost of about US$ 100 billion,^5^ however, their business case would accommodate twice that budget. Although Mars One is in part financed through money from donors across 100 countries and their numbers are growing, the donated money is not sufficient to fully finance the operation. As such, the non‐for‐profit arm of the business works closely with the for‐profit Mars One Ventures, the focus of which is to derive and maximize revenue from activities associated with the mission. These include sales of merchandise, brand partnerships, speaking engagements, and, once the mission is closer to the first human launch to Mars, broadcasting rights, Intellectual Property rights, entertainment content, and events. A portion of the proceeds from these revenue streams (as 5% of gross turnover) feed into the mission.^68^

It is evident that at present any potential revenue derived from the mission centers on selling the unique historic experience of sending humans to Mars, rather than from discovery and extraction of resources. There have been speculations by Mars colonization enthusiasts, such as Walker and Zubrin that it may be possible for Mars colony to become profitable by exploiting vast domestic resources of deuterium, which can be used as fuel for fusion reactors.^69^ Yet others, including Musk, argue that it is unlikely that Mars would offer anything material that would be financially viable to export to Earth.^70^

So, what might be the major benefit of Mars exploration? Should we not start by fixing our own planet and learning from this experience before attempting to conquer another outpost? Stratford tackles this notion from a different angle, and proposes to consider Mars colonization as a stimulus that is desperately needed by our contemporary society to move forward and once again regain our ability to tackle pressing problems head on:

*“We need an inspired generation to take fast action on so many fronts, but so far, our generation is not inspired. We have instead grown cynical and soft. Sending humans to Mars is the wildcard our world needs to change us from a stagnating, inward‐looking society into a problem solving, frontier‐looking society. It can be done now, and humans can be on Mars within the next ten to fifteen years. We just have to make that decision to go. If we can do this with Mars, this will be the first step forward for our society becoming a “can do” world. Let's take that step”*—writes *Frank Stratford*.^71^

## 5 Quo Vadis, the Only Civilization We Know?

Even among space enthusiasts, there is a rich diversity of opinions regarding “if,” “how” and “when” we should proceed with our space exploration and colonization ambitions. Unless we face a major cataclysm that would immediately threaten our existence on Earth, it is unlikely that a consensus on whether we need a Martian outpost would be reached any time soon. As it stands now, Mars One and similar projects are likely to continue, evolving and morphing as we learn more about the worlds beyond our own. As we gain new technological capabilities and grow our presence in the near‐Earth space, with both areas showing no sign of slowing down, we may be faced with moral and ethical challenges of sending humans to Mars far sooner than anticipated.

At present, it is challenging to comprehensively outline all related questions, let alone offer feasible solutions to these formidable challenges. The aim of this brief Essay is to introduce the interested reader to a vast range of arguments pro and contra Mars colonization, and many often contradictory and antilogous drivers for this project. This is not surprising for such a global challenge, and there is little doubt more questions will emerge, from shorter‐term “Would the colonists be representative of the global human population?” and “Who will finally decide who gets to go?” to longer reaching question around legal matters, the growth of Mars population and development of the social life on Mars.

Even the selection of the most proper “model of civilization” is still an open question. Indeed, there is no monolithic human civilization on Earth to mirror. Furthermore, establishment of societies of altruistic technologically savvy individuals may be far more challenging that it is anticipated. Indeed, with no relevant experience in building similar isolated, artificially built societies, the experience of polar investigators and long‐term space station expeditions, possibly complemented with the long‐term Moon station experience, will have to be used as the best available approximation for the self‐establishing, self‐organizing Mars colonies.

## Conflict of Interest

The authors declare no conflict of interest.
